# Methodology of Chip Temperature Measurement and Safety Machining Assessment in Dry Rough Milling of Magnesium Alloys Using Different Helix Angle Tools

**DOI:** 10.3390/ma17092063

**Published:** 2024-04-27

**Authors:** Ireneusz Zagórski, Piotr Zgórniak, Witold Habrat, José Machado, Stanisław Legutko

**Affiliations:** 1 Department of Production Engineering, Mechanical Engineering Faculty, Lublin University of Technology, Nadbystrzycka 36, 20-618 Lublin, Poland; 2 Institute of Machine Tools and Production Engineering, Faculty of Mechanical Engineering, Lodz University of Technology, Stefanowskiego 1/15, 90-537 Łódź, Poland; piotr.zgorniak@p.lodz.pl; 3 Department of Manufacturing and Automation Technologies, Rzeszow University of Technology, Powstańców Warszawy 12, 35-959 Rzeszów, Poland; witekhab@prz.edu.pl; 4 MEtRCs Research Center, Campus of Azurém, University of Minho, 4800-058 Guimarães, Portugal; jmachado@dem.uminho.pt; 5 Institute of Mechanical Technology, Faculty of Mechanical Engineering, Poznan University of Technology, Piotrowo 2, 61-138 Poznań, Poland; stanislaw.legutko@put.poznan.pl

**Keywords:** magnesium alloys, rough milling, temperature in cutting zone, chip temperature, infrared measurement, carbide tools, helix angle

## Abstract

This paper presents the methodology of measuring chip temperature in the cutting zone in the rough milling of magnesium alloys. Infrared measurements are taken to determine the effect of variable cutting speed, feed per tooth, and depth of cut on the maximum temperature of chips. Thermal images of chip temperature for a generated collective frame and corresponding histograms are presented. Chip temperatures are presented in numerical terms as median and average values; maximum and minimum values; range; and standard deviation. Box plots are also shown for selected machining conditions. The problems arising during signal recording with a mean emissivity coefficient ε = 0.13, a value which is dedicated during machining magnesium alloys, are discussed in detail. Chip temperatures obtained in the tests do not exceed approx. 420 °C. Therefore, the dry rough milling process carried out with carbide tools with different blade geometries can be considered safe for a wide range of machining parameters. The proposed methodology of chip temperature measurement and result processing is a new and effective approach to safety assessment in the dry milling of magnesium alloys.

## 1. Introduction

Despite the fact that the goal of any material removal process is to produce a component that meets exact specifications, safety requirements should not be underestimated, especially when cutting magnesium alloys. Milling is one of the most popular removal processes employed in the aerospace and automotive industries. Usually, parts are made of magnesium alloys to ensure reduced mass and satisfactory mechanical and heat-resistant properties [1]. The machining of these materials without a careful selection of cutting parameters can cause chip ignition. To ensure the required quality of a finished part and to improve productivity at the same time, one must know the temperature distribution in the cutting zone in order to select optimized cutting parameters [2,3]. In spite of its limitations, the use of thermal imaging systems for monitoring the cutting zone temperature seems to be an invaluable tool. In the literature one can find many studies on determining temperature in the machining of difficult-to-cut alloys, such as nickel and titanium alloys (e.g., Inconel) [4,5]. A very comprehensive overview of temperature measurement methods used in cutting processes has been given by researchers from the United States of America, Japan and Sweden [6]. Most of these studies deal with modelling and simulating different cutting processes—e.g., a model of intermittent cutting with specially created samples [5]—or manufacturing processes characterized by simpler kinematics, e.g., turning [4]. However, the measurement of cutting temperatures in the milling process pose unique technological challenges that are connected with complex kinematics and disturbing factors (e.g., different emissivity coefficients for components in the cutting zone and its heat-induced temperature, low values of emissivity coefficients for workpiece material and chips, rotation of the cutting tool, ambient conditions, disturbance from different heat sources, etc.). The most important limitation of IR measurement is the inability to use cooling lubricants during the cutting process. Although other methods of temperature measurement such as thermocouples and pyrometers allow for analysing fast-changing processes, they do not provide sufficient data for examining chip temperature in the cutting zone. Ueda et.al proposed a method for determining temperature on the flank face of the cutting tool in high-speed milling [7]. The method involved using a two-colour pyrometer and optical fibre inserted into the workpiece. Although thermocouples make it possible to measure temperature only at a specific point in the workpiece or tool, there is currently no other better measuring method for detecting changes in chip temperature in cutting processes. When analysing heat balance in milling processes for magnesium alloys, chip temperature deserves special attention. From the point of view of manufacturers, the issue of chip temperature seems completely irrelevant. More often, one wonders how much heat penetrates the workpiece and the cutting tool. In this respect, it is possible to predict possible phase changes affecting the strength of finished parts or to design the manufacturing process such that it takes into account the cutting tool’s service life [4]. Nevertheless, chips have the smallest volume and hence heat up the fastest. When cutting magnesium alloys, this may lead to chip ignition under unfavourable conditions, and in extreme cases, it may cause damage of equipment in production plants and workshops [8].

Table 1 lists selected results of machining conditions and research objects (machining indicators) for different grades of magnesium alloys. This compilation is primarily based on the publications devoted to the safety of machining magnesium alloys (e.g., cutting zone temperature, chip characteristics, and direct chip ignition during milling). Cutting zone temperature is a particularly important aspect of machining structural materials such as magnesium alloys [9,10,11,12,13,14,15,16,17,18,19,20,21,22,23,24], titanium alloys [25], nickel alloys, and steel alloys [26,27,28] with increased chromium content [3].

Interesting results were also obtained from studies investigating both the machinability of magnesium alloys and other fundamental properties connected with their flammability. These studies investigated the ignition point of given grades of magnesium alloy. For example, a study [16] investigated the effect of cerium (Ce) and aluminium (Al) addition in WE43, AZ31 and AZ91 alloys on their ignition point and oxidation resistance [17,18]. It was found that WE43 had the highest ignition point of 644 °C, compared to 628 °C for AZ31 and 600 °C for AZ91. Nevertheless, research studies on magnesium alloy chip ignition [19,22] seem to be of the greatest practical significance. Therefore, it seems equally important to explore the exact nature of chip fraction formation [29,30] and to describe machining safety in terms of specific chip fractions, their mass, and fragmentation [23].

It was already used in the 1990s simultaneous infrared and tool–chip thermocouple for temperature measurements in end-turning processes [31]. Various material groups were used in the presented research: steel, aluminium (2024), brass and grey cast iron. Similarly, ref. [32] used a thermal imaging infrared camera to measure the temperature of the cutting area during turning thin-walled Al-7075 workpiece. Moreover, artificial neural networks and adaptive neuro fuzzy inference systems were used to model and predict the temperature in turning. Infrared imaging was used again in [33] to analyse effective parameters which increase measurement accuracy (extracting and calibrating the emissivity coefficient for different temperature ranges). The tests were carried out again for turning a thin-walled workpiece (Al-7075 aluminium alloy). Chip temperature measurement can also be performed in the case of orthogonal cutting of the disc with 5754 aluminium alloy and S235JR steel [34]. The emissivity values were 0.2 for steel and 0.3 for oxidized aluminium. Measurements using an infrared camera are also carried out for other material groups. A good example is the material group of titanium alloys, where the Ti–6Al–4V alloy is most often used in research [35,36,37,38]. It is interesting to use transparent yttrium aluminium garnet tools for turning titanium disc to observe the area of the tool–chip interface (through these tool) and measures the temperature distribution [35]. Similarly, orthogonal cutting tests of Ti6Al4V alloy wit using infrared measurements with high resolution were performed in [36]. Measurements of the temperature in the shear zone were made. Therefore, even in very modern measurements using the newly developed near-infrared two-colour pyrometer system [37], the cutting zone temperature was measured only for turning processes. The developed pyrometer can be used to measure tool edge temperature and chip temperature in dry and wet cutting conditions.

Few works concern the measurement of cutting temperature during milling. This is due to difficult temperature measurement during high dynamics of interactions in the cutting zone. An example of re-using a high-speed infrared camera is an attempt to estimate the temperature of the cutting edge and chip temperature during milling Ti-6Al-4V [38]. The tool–chip contact zone has small dimensions of 8 × 5 mm and an integration time of 1µs (for full frame). It was observed that the measured cutting temperature is about 450 °C.

Oliveira Moreira et al. [39] determined the emissivity coefficient through an experimental method that took into account both the directional spectral and diffusive grey approaches to the estimation of emissivity. The emissivity coefficient was a key parameter in the temperature estimation process, and its accurate determination was necessary to obtain reliable results. The authors emphasized that emissivity may vary significantly depending on the material, its surface condition, and temperature, which required a detailed analysis and adaptation of the measurement methodology to specific experimental conditions. Guimarãesa et al. [40] used two main methods for measuring temperature in the cutting zone: the thermocouple method built into the cutting insert and infrared thermography (IR). They showed that the embedded thermocouple method allows the obtaining of useful information for optimizing cutting parameters, which can contribute to increasing productivity and tool life. The maximum temperature measurement error using this method was 0.96%. The authors noted that although IR thermography showed the same trend as the built-in thermocouple, the temperatures it measured were always lower. This was due to measurement difficulties such as interfering chips and possible underestimation of emissivity values, which led to lower temperature readings by the thermography camera. Karaguzela et al. [41] presented a new experimental technique for measuring temperatures during face milling, which was used to verify the developed analytical model. The work noted that measuring the cutting temperature in the milling process is more difficult than turning due to experimental difficulties and transient characteristics of the process. Grochalski and Jabłoński [42] also discussed two methods of measuring the temperature of the tool tip, but during turning–measurement with a thermocouple built into the tool and the thermal imaging method. The authors concluded that both methods provided consistent temperature values, with the largest difference between the specified processing parameters not exceeding 11 °C. They also noted that the thermal imaging method can provide more measurement data but is more sensitive to interference, which significantly limits its application. Liu et al. [43] focused on the study of cutting temperature in the side milling process. They emphasized the importance of monitoring the temperature of the cutting tool due to its impact on the chip formation mechanism, tool wear, tool life, surface quality and machining tolerances. They used advanced methods of temperature measurement in the cutting zone, which were based on continuous measurement of transient temperature using wireless transmission technology.

Cichosz et al. [44] presented methods used to measure the cutting temperature. They discuss both the advantages and disadvantages of individual methods, as well as their usefulness in various types of machining processes. They also indicate the possibility of methodological errors that may significantly reduce the accuracy of the measurements. They draw attention to the difficulties associated with identifying the cutting temperature, which result from the limited volume of the cutting zone, high temperature gradients, differences in the thermal conductivity of tool materials and workpieces, as well as interference caused by anti-abrasive layers, cutting fluids, and the movement of heat-emitting surfaces.

Also important from the point of view of the risk of ignition during machining –re the so-called temperature and ignition point for a given grade of magnesium alloy. In general, it can be assumed that the values considered dangerous during machining are those when the chip temperature exceeds approximately 500 °C. In the case of the AZ91 alloy, the literature details the following temperature values defined as the so-called ignition point: Lin et al. [16]—525 °C; Liu et al. [17]—600 °C; Ravi Kumar et al. [18]—580–590 °C. These differences may occur due to the details of the test performed.

This paper presents a new approach to the analysis of temperature fields from sequences of thermal images obtained with the FLIR SC 6000HS (Wilsonville, OR, USA) thermal imaging camera. For this purpose, a special algorithm was developed in the MATLAB program (The MathWorks, Inc., Natick, MA, USA), which involves creating a collective frame that takes into account maximum chip temperatures from the cutting process, from the entry of the cutting tool into the workpiece until the end of chip formation. The innovation of the work lies in its comprehensive approach to examining the influence of tool geometry, in particular the helix angle, on chip temperature and, consequently, the safety of dry machining of magnesium alloys. This work provides new information on key aspects of the cutting process, offering practical tips to optimise the machining parameters for increased safety and efficiency. The helix angle of the tool can significantly affect cutting efficiency, heat generation, and the risk of chip ignition, which is crucial when machining flammable materials, such as magnesium alloy. The methodology to measure the chip temperature in the cutting zone during dry machining is also an element of innovation. The method of determining the emission coefficient using a tube furnace and the analysis of the results allowed accurate tracking of temperature changes, which is necessary to assess the safety of the process. These studies take into account not only technical aspects such as chip temperature and tool geometry but also potential threats to process safety. Additionally, taking into account the analysis of the impact of various technological parameters, such as cutting speed, feed per tooth, or cutting depth, on chip temperature allows the identification of optimal machining conditions, minimizing the risk of ignition and increasing the efficiency of the process.

## 2. Materials and Methods

**Figure 1:**
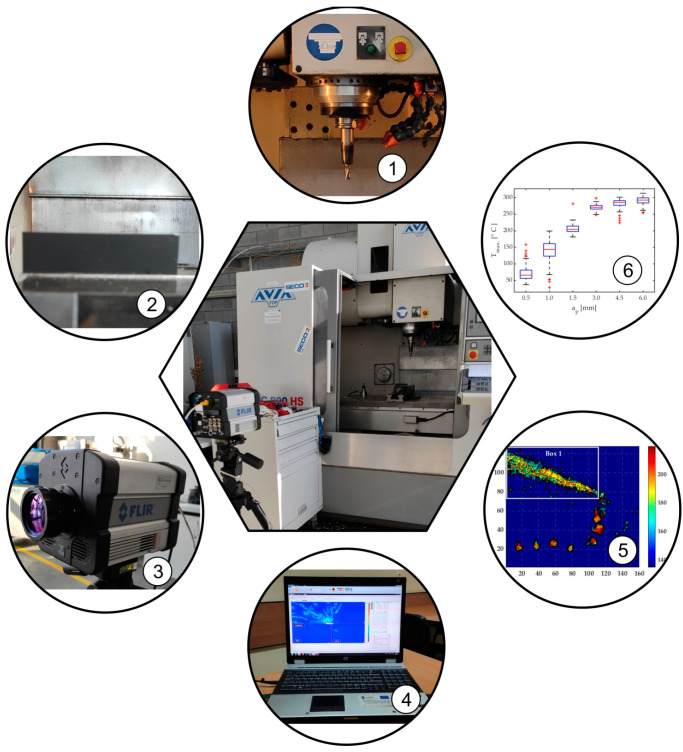
The experimental stand: 1—cutting tool, 2—workpiece, 3—IR camera FLIR SC6000HS, 4—computer with FLIR Research IR 4.20.2.74 64-bit software, 5—IR frame generated by MATLAB R2022a software, 6—analysis of the final results in the form of a box plot.

Infrared measurements were made with a FLIR SC 6000 HS thermal camera. The distance between the camera and the analysed object was 2.14 m. The camera had a standard lens with a focal length of 50 mm and F/2.3 diaphragm, covering the wavelengths ranging from 3 µm to 5 µm. The experimental stand is presented in Figure 1.

The distance between the camera position and the recorded object was selected in such a way that the front surface of the workpiece and the tool were in the area of the highest possible sharpness (field of focus). Then, this distance was measured and entered into the thermal imaging camera FLIR SC 6000 HS thermal camera software in order to correctly select the conditions in which the measurements were carried out. The value of the average emissivity coefficient was experimentally determined for two magnesium alloys used in the presented research. These values were entered into the FLIR Research IR 4.20.2.74 64-bit software (Wilsonville, OR, USA). The study was conducted on test specimens made of two different grades of magnesium alloy: AZ91D and AZ31B. For these materials, the average emissivity coefficient values were 0.31 and 0.24, respectively, and were changed in the data analysis field after recording all sequences. For temperatures in the range of 150 to 350 °C, the change in emissivity coefficients for individual materials did not exceed 10%. The value adopted during data acquisition concerned the emissivity of the sample painted black and was 0.92. The correctness of the obtained temperature values was also verified by placing a black soldering tip at the set temperature in the measurement zone. The results obtained in this way confirmed the correctness of the assumptions made for the sample material. In the case of recording chip temperatures, the matter was not so clear because after the chip was separated by the cutting tool, it changed its position and rotated and changed its position unpredictably. Moreover, the value of the emissivity coefficient could not be assumed at the level of 0.92 assumed for the black sample, because the chips had a shiny surface without the black colour. This fact forced the authors to take the trouble to determine the values of emission coefficients at a separate site in an experimental manner. Since the research focused on determining the maximum temperatures of chips, the values recorded by the camera were also influenced by the position of the chips and their distance from the area of focus. By focusing on the front surface of the sample, it was possible to record both sample and chip temperatures. However, in this case, the number of chips that appeared in the focus was random. It was decided that using the highest possible frequency of recording thermal images would minimize the unfavourable effect of the variability of the number of chips in the sharpness zone and contribute to higher chip temperatures being recorded in a larger number of frames.

**Figure 2:**
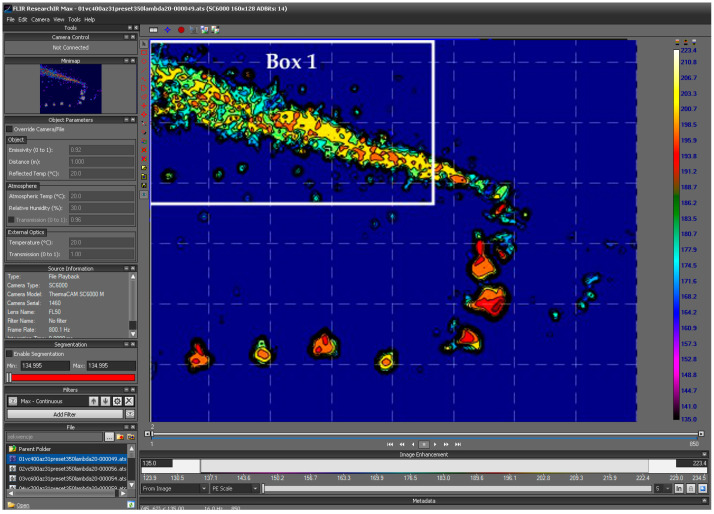
View of the FLIR Research IR graphical interface.

**Figure 3:**
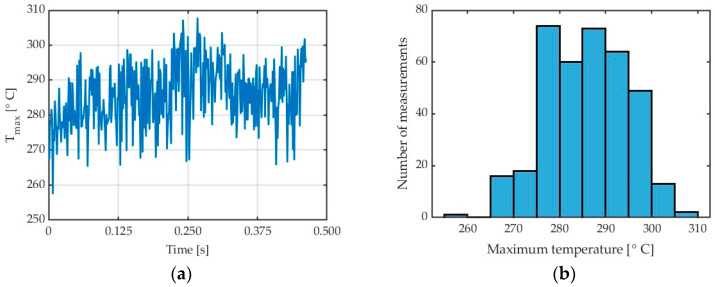
AZ31B alloy: (**a**) chip temperature for a generated collective frame; (**b**) histogram, in a cutting process conducted with v_c_ = 400 m/min, λ_s_ = 20°, ε = 0.31.

The study involved analysing the temperatures generated by the chips in the cutting zone without taking into account the temperatures of the workpiece and cutting tool. Due to rapid changes in the chip temperature and variations in the cutting parameters, it was necessary to carry out preliminary tests in which chip temperature was measured using different camera calibration ranges. Signals obtained from the tests conducted with extreme machining parameters were then analysed. Obtained signal values were used to determine a single calibrated range allowing signal measurement without the danger of signal saturation and thus signal cut-off. According to this procedure, one temperature preset was used to capture data in the tests from 150 to 350 °C. To ensure the largest possible data set, thermal imagery for both presets was captured with the highest possible frequency of 800 Hz. Authors performed acquisition of each test with two standard ranges of the IR camera. The first one, which might be useful for lower temperature values, like workpiece temperature, considers the range from 10 to 90 °C. The second one has been used for chip temperature evaluation ranging from 150 to 350 °C. The scope of this publication does not include workpiece temperature results, so a higher preset was adequate for chip temperature investigation. When recording a sequence of thermal photos, it was not decided to use the dynamic range extension function (DRX) because it resulted in a reduction in the resultant frequency of recording thermal photos. The use of all available active presets to extend the measurement range was associated with various recording parameters, such as longer integration times for lower temperature ranges. In this case, comparing temperatures in fast-changing processes, such as the milling process, would be influenced by the different numbers of frames recorded at different ranges and at different frequencies. It would not be possible to determine a constant recording frequency in all cutting tests. Based on the preliminary tests carried out, it was determined that taking into account dynamic range expansion from four active standard presets would reduce the possible recording frequency to approximately 100 Hz. Moreover, you would never know how many frames would be saved for each cutting test. Due to this fact, it was considered justified to select individual standard presets and possibly reduce the resolution of recorded photos in order to obtain the highest possible frequency. Taking into account the possibility of high temperature gradients in individual tests, it was decided to record images in two standard sub-ranges characterized by short integration times. Unfortunately, the standard range from 50 to 150 degrees did not allow for recording thermal photos with a frequency of 800 Hz. The aspect of selecting the highest possible recording frequency was closely related to the variability of the number of frames recorded for higher values of cutting parameters, i.e., feed per tooth and cutting speed. The shorter the cutting time, the fewer data there are for subsequent analysis of the influence of individual cutting parameters. For this reason, it was decided not to use the function of dynamically expanding the camera’s measurement range. On the other hand, the camera used during the tests was not specially calibrated for the extended measurement range. This option was an additional fee, and currently, the authors were not able to use it. Determining the chip temperature involved determining the analysis field in the form of a rectangle, for which the emissivity coefficient appropriate for the processed material was adopted. This field only included the area to the left of the cutting tool and above the workpiece in Figure 2. For each test, due to the fact that the sample size increased according to the material processed in the previous test, necessary adjustments were made to the analysis field in such a way that it covered the largest possible surface. Then, the Research IR program function was set to find the highest temperature in the analysis field and these data were recorded as a file with the *.csv extension. Line charts of the maximum chip temperature in the recorded frames were made based on these data. An example of this type of chart is shown in Figure 3a.

Consequently, the examined area had to be reduced to 128 × 160 pixels. 800 frames were recorded per every test, which corresponded to a cutting time of 1 s. After acquisition, during data mining, these time periods have been corrected to get only appropriate data values without lower temperatures at the start and the end of recorded test’s sequences.

The radial depth of cut a_e_ was maintained constant at 14 mm. The axial depth of cut a_p_ was variable and ranged from 0.5 to 6 mm depending on the test. Other variables were: cutting speed v_c_ (from 400 to 1200 m/min) and feed per tooth f_z_ (from 0.05 mm/tooth to 0.3 mm/tooth). Carbide tools with a 16 mm diameter and a variable helix angle λ_s_ = 20° and λ_s_ = 50° were used. They were mounted in CELSIO HSK-A63 toolholders with a balance grade of G2.5 (in compliance with ISO 21940–11:2016 [45]). The residual imbalances were 1.41 gmm (λ_s_ = 20°) and 1.24 gmm (λ_s_ = 50°), respectively.

The study was conducted on test specimens made of two different grades of magnesium alloy: AZ91D and AZ31B. Preliminary research was divided into two stages. In the first stage of the study, maximum chip temperature was analysed in compliance with recommendations given in the literature [46], according to which the mean emissivity coefficient of magnesium alloys is ε = 0.13. Initially, a soldering tool with temperature control was put in the cutting zone. However, the estimated temperatures were so high that it was decided to estimate emissivity coefficients in an experimental way. The emissivity coefficients were determined based on calibration using a laboratory tube furnace. Samples of the tested materials were placed in the central point of the furnace and heated from 150 to 350 degrees Celsius in 50-degree increments until stabilization. Above this temperature, oxidation appeared on the surface, which distorted the results. The samples were observed with a thermal imaging camera and the emissivity coefficient was determined based on the stabilized temperature. On the base of experiments performed for two types of magnesium alloys, two values of emissivity coefficients have been estimated. For two investigated workpiece materials, the average values have been taken into consideration accordingly: for AZ31B emissivity coefficient, ε = 0.31; for AZ91D, ε = 0.24. This simplification seemed justified for comparative purposes, which is the case in this study. Thermal imagery was taken by FLIR’s ResearchIR [47]. A view of the program’s graphical interface is shown in Figure 2.

The procedure for determining chip temperatures included utilizing a filter that used a Matlab script for identifying and acquiring maximum chip temperatures in successive frames. The script recorded the highest temperatures from successive frames of thermal imagery sequences on a single collective frame, which was later used to compare the effects of individual cutting parameters, workpiece material and cutting tool.

Based on all frames acquired in the test, a collective linear graph was generated. It presents maximum temperature detected into analysis box vs. frame number. Due to the differences in chip form and the direct presence of the cutting tool, the temperatures were analysed in a data analysis box, as marked in Figure 2 with a white rectangular area and named Box 1. In all tests, the locations of this area were updated for each test to show area without cutting tool and workpiece. The linear collective graph was then exported as a file in the (*.csv) format, which allowed further analysis in Matlab.

During the acquisition of thermal imagery, it was observed that the ranges of calibrated temperature had to be changed, which resulted from different values of heat generated during milling. This fact made it necessary to develop a method for processing measurement data.

The determination of the effect of variable emissivity coefficient with increasing temperature in the cutting zone proved to be a very difficult problem, requiring the use of specialized equipment which the authors of this study did not have at their disposal. In addition to that, when determining chip temperature, the authors had no control over chip position recorded in the frame.

## 3. Results

Figure 3 shows the diagrams illustrating maximum chip temperature in the cutting zone. Figure 3a shows the so-called collective linear graph with all maximum chip temperatures obtained in the entire cutting process from the moment when the tool would cut into the workpiece until the end of chip formation with the applied machining parameters. It must be stressed that the analysis was not performed for the entire frame but only for a region where chips would appear (Figure 2 and Figure 3a).

Figure 3b shows the so-called histogram illustrating a relationship between the maximum temperature and the number of measurements. It can easily be observed that the most numerous group of results (about 74) is the temperature values, ranging from 275 to 280 °C. An analysis of individual frames reveals the presence of the highest temperatures in the final stage of the cutting process.

Table 2 gives detailed results of the cutting process for AZ31B alloy, conducted with a cutting speed v_c_ = 400 m/min and tool helix angle λ_s_ = 20°. The mean emissivity coefficient of ε = 0.31 was used in this process.

**Figure 4:**
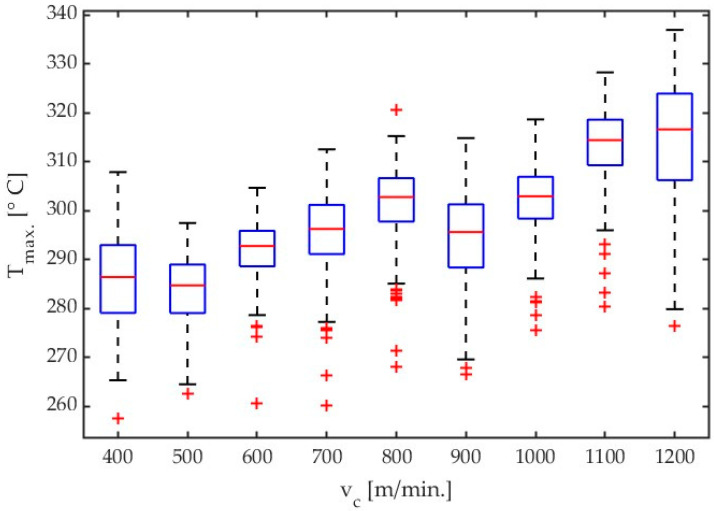
Box plot obtained for AZ31B alloy with an emissivity coefficient ε = 0.31 for all tests concerning influence of cutting velocity. Tool with λ_s_ = 20°.

Figure 4 shows the box plots comparing the effect of variable cutting speed. The results are given for AZ31B alloy and a helix angle of λ_s_ = 20°.

Based on “saw shaped” charts showing the dependence of the maximum temperatures detected in the analysis field Figure 2, boxplots were constructed for subsequent cutting tests. An example of which can be seen in Figure 4. These charts were generated using scripts written in Matlab software. The values presented in the boxplots concern the maximum temperatures determined in subsequent frames from the moment the tool enters the workpiece until the last chip is separated, i.e., the end of the cutting process. As can be seen in Figure 3a, the following frames had variable values of maximum chip temperatures. On the base of data obtained in this way, it was possible to determine which maximum temperature determined from the data analysis field was obtained in the entire sequence of thermal photos for a specific test. The median and average range were determined in the same way. Knowledge about creating box plots is generally available—for example, as Internet resources or in the documentation of the Matlab program—and was not presented in the content of the article.

**Figure 5:**
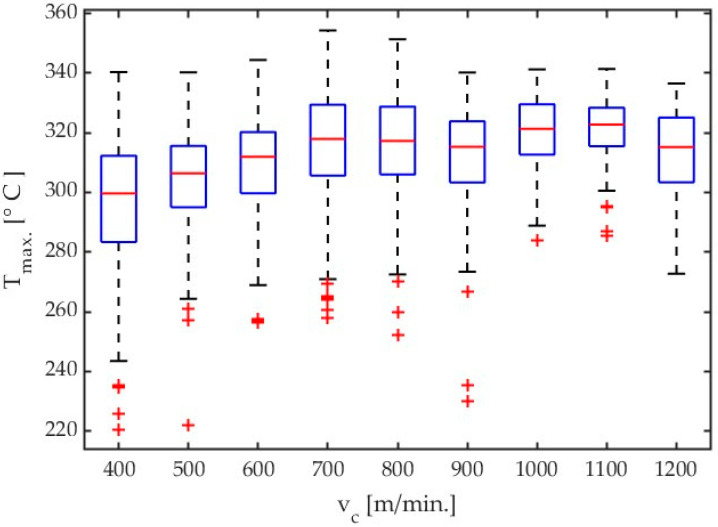
Box plot obtained for AZ31B alloy with an emissivity coefficient ε = 0.31 for all tests concerning influence of cutting velocity. Tool with λ_s_ = 50°.

**Figure 6:**
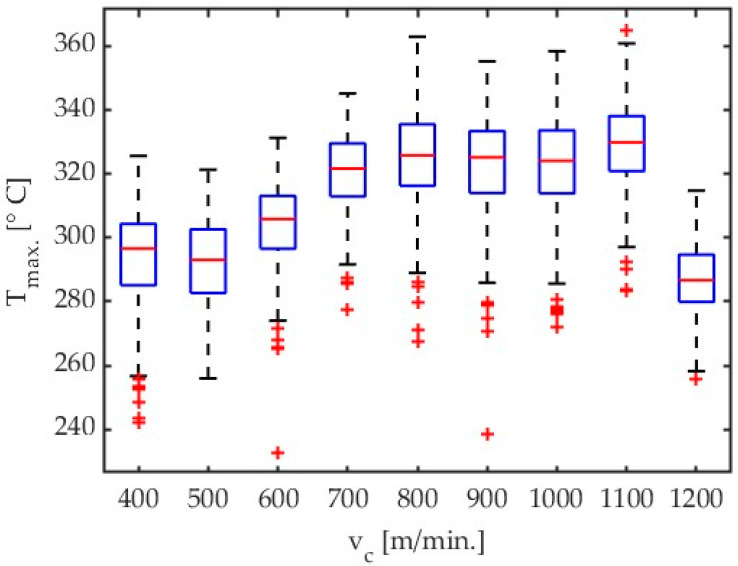
Box plot obtained for AZ91D alloy with an emissivity coefficient ε = 0.24 for all tests concerning influence of cutting velocity. Tool with λ_s_ = 20°.

**Figure 7:**
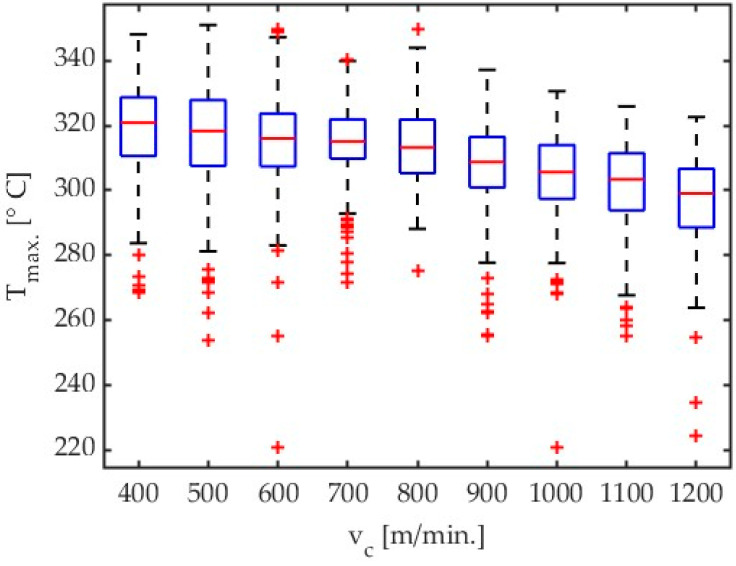
Box plot obtained for AZ91D alloy with an emissivity coefficient ε = 0.24 for all tests concerning influence of cutting velocity. Tool with λ_s_ = 50°.

An analysis of the results given in Table 4 and Figure 4 demonstrates that a signal for the highest temperatures mainly increases with an increase in cutting velocity with almost all tested cutting speed values, except for the lowest one, i.e., v_c_ = 400 m/min. Results presented in Figure 4, Figure 5, Figure 6, and Figure 7 show the influence of cutting velocity on maximum temperatures for different workpiece materials (AZ31B, AZ91D) and different cutting tool geometries (λ_s_ = 20°, λ_s_ = 50°) in the form of boxplots.

**Figure 8:**
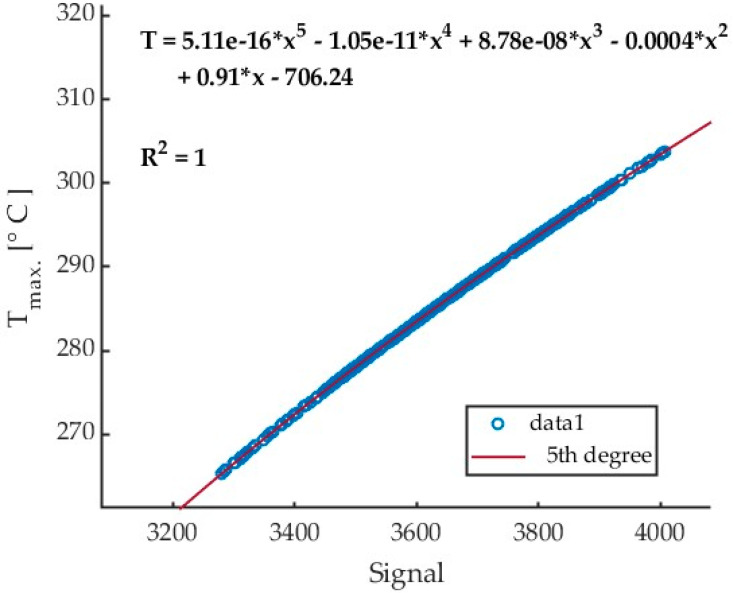
Calibration curve for test 1 (v_c_ = 400 m/min, AZ31B, angle λ_s_ = 20°) with 5th degree polynomial fitting relationship.

An example of a calibration curve obtained from Test 1 in cutting AZ31B magnesium alloy with a cutting speed v_c_ = 400 m/min using a tool with a helix angle of λ_s_ = 20° is presented in Figure 8. The curve was generated for every collective frame separately for each test. The same frame was recorded for both signal and temperature values. The values of the same pixels were then compared, and a calibration curve was generated. For Tests 1 through 9, all data were within the calibrated range, and there was no need to recalculate the signal values exceeding the calibration range (temperatures below 350 °C).

An analysis of the data in Table 2 for the maximum chip temperature of 307.88 °C in test 1 reveals that all temperatures were calculated by a fifth-order polynomial. Table 3 gives detailed results of the cutting process for AZ31B alloy, conducted with a cutting speed v_c_ = 1200 m/min and tool helix angle λ_s_ = 20°.

In the above two cases, the observed maximum temperatures do not exceed 350 °C. However, by increasing the cutting speed, we obtained a threefold increase in machining efficiency and productivity without causing any significant increase in the cutting zone temperature—the temperature increased from 307.88 °C to 336.97 °C, which was about 10% of the maximum value. The observed temperatures can therefore be considered safe, and—consequently—the machining range can, too, be defined as safe.

**Figure 9:**
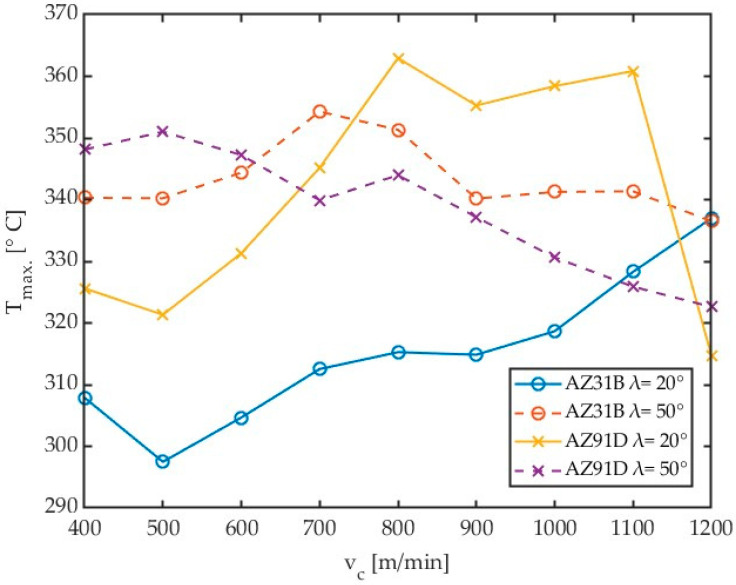
Comparison of the effect of cutting speed for different magnesium alloys and tools.

The above methodology was employed to investigate the effect of variable cutting speed on the maximum chip temperature in milling AZ31B and AZ91D magnesium alloys by tools with variable helix angle λ_s_. Results obtained for different workpiece materials and helix angles are given in Figure 9.

An analysis of the data in Figure 9 demonstrates that the highest chip temperatures were achieved for AZ91D alloy machined using a tool with a helix angle of λ_s_ = 20°. For this material, an increase in the helix angle for lower cutting velocities caused an increase in chip temperature. However, for greater cutting velocities, the opposite effect has been observed. Interestingly, for most cases, lower temperatures were obtained with the highest tested cutting speed, which is a positive effect of machining and may indicate both the stability of the process conducted with the highest cutting speed as well as the “activation” of the HSM process. The data shown in Figure 9 are listed in Table 4.

**Figure 10:**
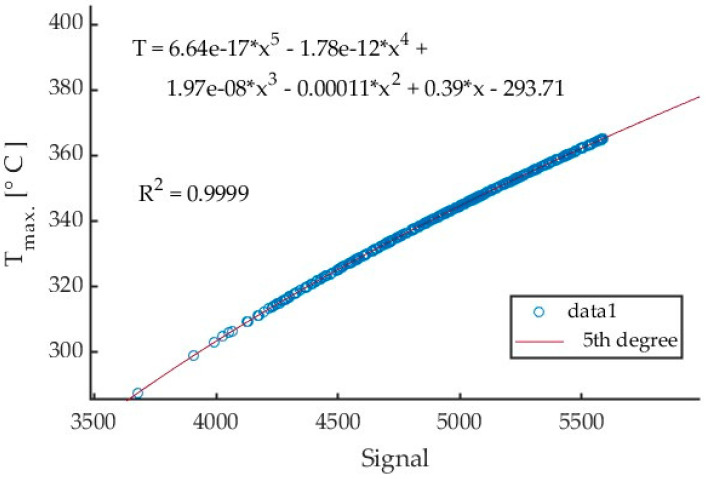
Calibration curve for Test 10 (f_z_ = 0.05 mm/tooth, AZ31B, λ_s_ = 20°) with 5th-degree polynomial fitting relationship.

Results of the tests investigating the effect of changing the feed per tooth from 0.05 mm/tooth to 0.30 mm/tooth on the maximum chip temperature can be compared. All tests were conducted for a calibration range from 150 to 350 °C. An example of a curve generated for Test 10 is presented in Figure 10.

**Figure 11:**
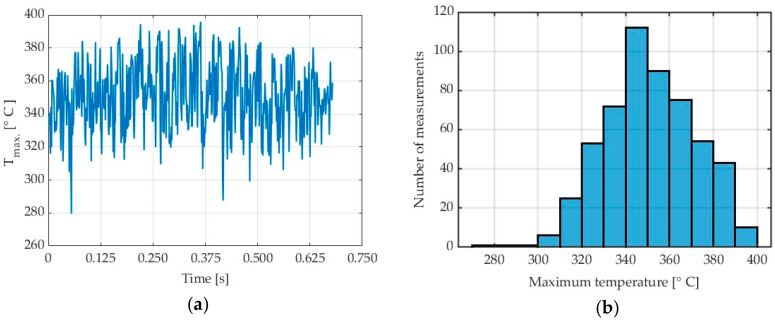
AZ31B alloy: (**a**) chip temperature for a generated collective frame; (**b**) histogram, in a cutting process conducted with f_z_ = 0.05 mm/tooth, λ_s_ = 20°, ε = 0.31.

Figure 11a,b show the chip temperatures obtained from Test 10 for a generated collective linear graph and a histogram in a cutting process conducted with a feed per blade of f_z_ = 0.05 mm/tooth and a modified emissivity coefficient of ε = 0.31.

**Figure 12:**
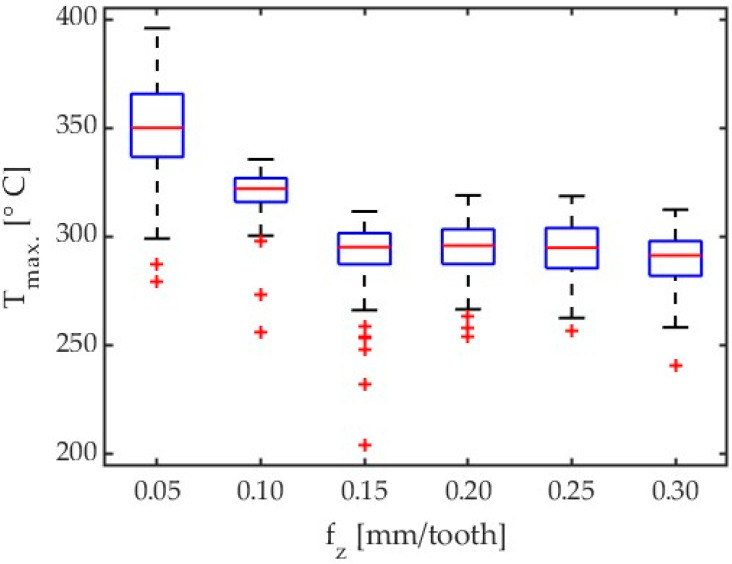
Comparison of the effect of feed per tooth on chip temperature for all tests for AZ31B and λ_s_ = 20°.

An exemplary boxplot for AZ31B magnesium alloy with λ_s_ = 20° shows an influence of feed on maximum chip temperature is presented in Figure 12.

On the base of Figure 12 the maximum temperatures for lower feed per tooth has been observed. It might be connected with smaller cross-section of the chips. In this case the smaller heat capacity of the chip causes greater signal values. Table 5 gives detailed results of the cutting process for AZ31B alloy, conducted with a feed per tooth f_z_ = 0.05 mm/tooth and tool helix angle λ_s_ = 20°.

The above-described methodology was used to investigate the effect of feed per tooth on maximum chip temperature in milling AZ31B and AZ91D alloys conducted using tools with helix angles of λ_s_ = 20° and λ_s_ = 50°.

**Figure 13:**
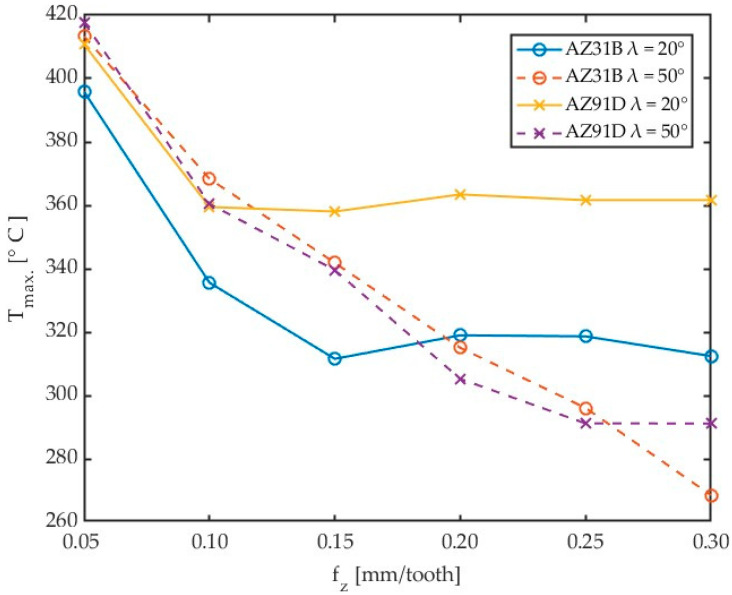
Comparison of the effect of feed per tooth on chip temperature for different magnesium alloys and tools.

Results showing the effect of different workpiece materials and helix angles are presented in Figure 13.

For the analysed feed per tooth range, one can observe that chip temperature generally decreases with increasing feed per tooth. The maximum chip temperature decreasing with increasing the feed per tooth can be explained by milling process dynamics and constant frequency of thermal imagery acquisition, as these two factors can affect the emitted thermal energy value measured by the thermal camera. Moreover, as the feed per tooth is increased, the testing time becomes shorter, which also affects the number of analysed data points. This relationship can be traced in the histograms. In further studies, it would be useful to investigate temperatures that are predominant in a given test, besides of maximum temperatures. To give an example, for Test 10 for AZ31B and λ_s_ = 20°, instead of the maximum temperature (395.86 °C), one should perhaps consider a temperature of 332.24 °C, which occurs most often in the data analysis field, as shown in Figure 11b. Nonetheless, as milling safety depends on the maximum temperature of chips, in this study, we analyse the temperatures referred to as T_max_. The data given in Figure 13 are presented in detail in Table 6.

Another parameter that was analysed was the axial depth of cut a_p_. For the tests conducted with the depth of cut a_p_ ranging between 0.5 mm and 6 mm, it was observed that for low depths of cut, the signals recorded for the 350 preset were insufficient to obtain temperatures from the calibrated curve range. To remedy this problem, it was necessary to recalculate lower signal values by employing extrapolated calibration curve equation to obtain lower temperatures than 135 °C.

**Figure 14:**
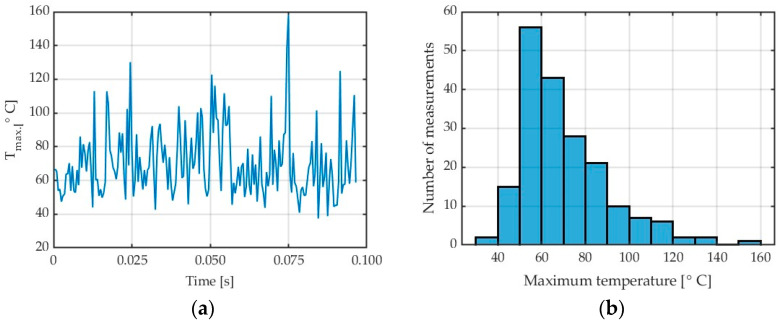
AZ31B alloy: (**a**) chip temperature for a generated collective linear frame; (**b**) histogram, in a cutting process conducted with a_p_ = 0.5 mm, λ_s_ = 20°, ε = 0.31.

An example of a collective linear frame obtained in Test 16 conducted with the variable depth of cut a_p_ = 0.5 mm for AZ31B magnesium alloy and tool helix angle λ_s_ = 20° is shown in Figure 14a. The histogram of test 16 is shown in Figure 14b.

Table 7 gives the detailed results obtained for AZ31B alloy in a cutting process conducted with a_p_ = 0.5 mm and a helix angle of λ_s_ = 20°.

**Figure 15:**
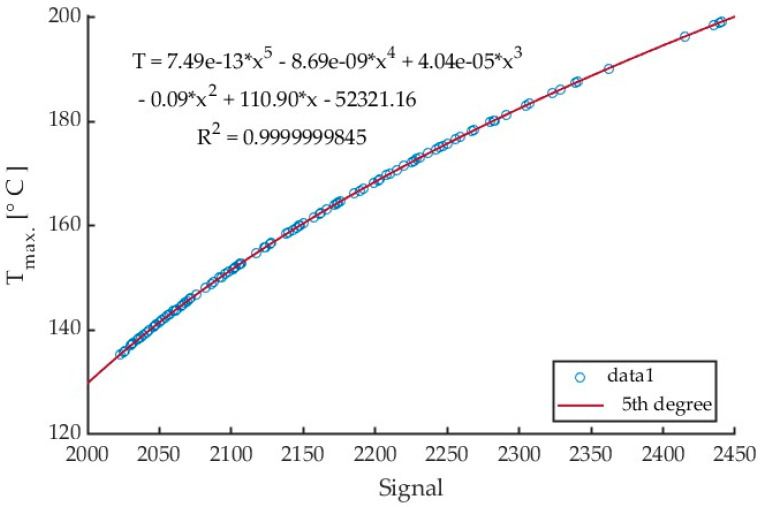
Fifth-degree polynomial calibration curve for test 17 corresponding to a_p_ = 1.0 mm, λ_s_ = 20°, ε = 0.31.

Based on the collective linear frames, a fifth-degree polynomial calibration equations have been obtained. The exemplary curve for Test 17 is shown in Figure 15.

**Figure 16:**
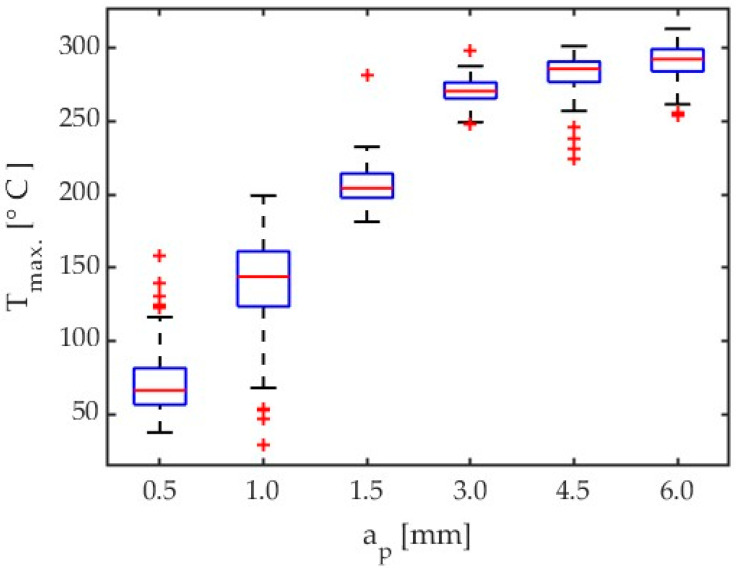
Chip temperature comparison for depth of cut influence of AZ31B, λ_s_ = 20°, ε = 0.31.

Figure 16 shows the boxplot for depth of cut results comparison for all tests concerning AZ31B magnesium alloy and a tool helix angle of λ_s_ = 20°.

Another step in this study was to validate the developed procedure for other tests by taking into account the variable depth of cut. For process automation, all scripts and relevant functions were generated in the Matlab software.

**Figure 17:**
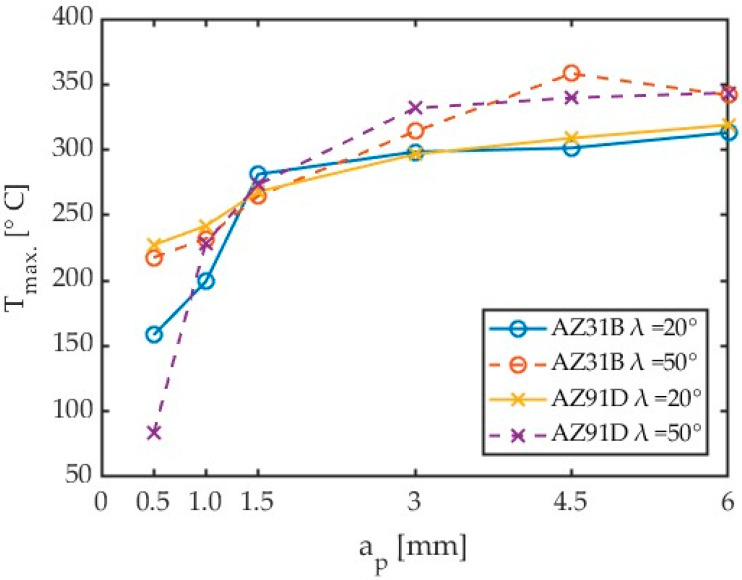
Comparison of the effect of axial depth of cut on chip temperature for different magnesium alloys and tools.

Tests from 16 through 21 for the 150 ÷ 350 °C preset have been taken into consideration for depth of cut influence on maximum chip temperatures into cutting zone. Some results, especially with lower values of depth of cut need to be extrapolated by means of 5th polynomial calibration curves for temperatures lower than 135 °C. The preset named 150 ÷ 350 °C achieved the lowest temperature close to 135 °C. However, it must be expected that these results are subject to greater error. In addition, the results of the tests conducted with low depths of cut are characterized by small amounts of data. The depth of cut results obtained for different materials and tools are plotted in Figure 17. A comparison of these results is given in Table 8.

Given the fact that the results produced by different algorithms had to be combined, the obtained depth of cut results should probably be verified by repeating the test using a different preset (e.g., 50 ÷ 150 °C), particularly for low depths of cut. However, an analysis of the results obtained with the 150 ÷ 350 °C preset for all depths of cut tests generally demonstrates that an increase in the depth of cut causes an increase in the chip temperature.

## 4. Discussion

The objective of this study was to determine the maximum chip temperatures in milling processes for magnesium alloys. In previous studies [3,20,21,23,24], different approaches were adopted for comparing chip temperatures in the cutting zone. The simplest one involved determining workpiece temperatures [2] where the temperature gradient was relatively low. This approach did not, however, involve examining cutting tool temperatures and chip temperatures. Given the relatively low temperatures generated in the workpiece and the fixed position of the workpiece relative to the optical system of the thermal imaging camera, it was possible to use lower frequencies for thermal imagery acquisition. Specifically, it was possible to use a dynamic extension of the temperature range into four calibrated presets. In effect, the camera loop recorded frames for each preset from the ambient temperature up to 350 °C. This approach made it possible to take measurements with a maximum frequency of 100 Hz. When the temperatures generated in the workpiece exceeded the calibrated range of the camera, the nearest frame to a higher preset could be examined. The procedure consisted of selecting representative frames that were located in the middle of the sampling length. This assessment method involved manual selection of a frame which was then compared to similar frames selected for other cutting parameters. The reproducibility and correctness of obtained results depended on the correct frame selection. This was by no means an easy process, as the position of the cutting tool in the middle of the sample was variable in the experiments (due to the effect of feed per tooth and cutting speed). Therefore, a choice had to be made as to which frame was the most representative.

The data acquisition process is much more difficult to perform when it comes to chip temperature analysis. Due to the fact that chip motion in the machine tool space is unpredictable in terms of chip position in frame, it was necessary to use the highest possible frequency of thermal imagery acquisition so that the moving chip could be recorded in the best possible focus and as close to the cutting tool as possible. In this position, the chip had the highest temperature. The formation of chips, their shape, as well as direction and position in the frame were variable depending on the cutting parameters and the chip formation time. The number of chips in the cutting zone was variable as well. Instead of using very tedious and time-consuming manual analyses as in previous studies [20,21,23,24], the authors decided to employ a procedure which involved generating a collective frame encompassing maximum chip temperatures from all frames from the moment of tool entry into the workpiece until the end of chip formation. This approach significantly facilitated the assessment of the impact of individual cutting parameters. A single frame took into account both the heating process (the beginning of the cutting process until the tool and workpiece temperatures became steady) and the steady-state chip temperature, which—for samples of the same dimensions—did not cause differences in maximum chip temperature obtained in individual tests performed with different cutting parameters. Additionally, the chip position in the cutting zone was of lesser importance since these were maximum chip temperatures from individual frames that were recorded. Specifically, the generated frame contained data from thousands of frames recorded in each test. Despite many positive aspects of this approach, data processing revealed that the number of temperatures recorded in the collective frame strictly depended on the cutting parameters. With higher cutting speeds and feeds per tooth, the number of frames used to generate a collective frame was reduced. This fact may have affected the correctness of the conclusions about maximum chip temperatures. As a result of using a fixed calibrated temperature range to ensure the maximum frequency of thermal imagery acquisition, data could not be acquired at lower temperatures due to the fact that the signal recorded by the thermal imaging camera was too weak. An analysis of the histograms demonstrated that the number of maximum temperatures was relatively small compared to that of most frequent temperatures.

It is difficult to compare the results reported in [9,12,14] with those presented in this study, as the previous studies were conducted with low values of axial depth of cut a_p_.

The results presented of this study may be compared to those reported in [20,21,23,24]. In these studies, rough milling was investigated and chip temperature in the cutting zone was measured on the chip surface.

When analysing the impact of cutting speed on chip temperature, two pairs of tools can be distinguished, with different shapes depending on the tool geometry. In the case of an angle of λ_s_ = 50° up to a speed of 800 m/min, the temperatures for both alloys are similar. A further increase in cutting speed causes a slight decrease in temperature. In the case of the analysed tool geometry, the use of high cutting speeds is safe due to the risk of ignition.

With the reduction of the angle λ_s_ to 20°, after a slight drop in temperature for v_c_ = 500 m/min, a significant increase in temperature occurs with the increase in cutting speed. In the case of the AZ91D alloy, the temperature stabilizes in the range from 800 to 1100 m/min, after which the temperature lowering effect known from the literature for high-speed machining is visible. For the AZ31B alloy and an angle of λ_s_ = 20°, this effect was not observed in the adopted temperature rang.

It follows that the use of HSM machining in magnesium alloys for the cutter helix angle λ_s_ = 50° is safer due to the decreasing tendency of temperature with increasing cutting speed, even at lower cutting speeds. Additionally, in [20], in the case of a cutter with a Kordell geometry and an angle of λ_s_ = 40°, a similar tendency was observed. Conversely, a drop in temperature with an increase in cutting speed for the analysed alloys was not observed for carbide tools with TiAlN coating [21] and a PCD blade [23].

It can be predicted that for the analysed tools with an angle of λ_s_ = 20°, the effect of temperature reduction with increasing cutting speed may occur for higher cutting speed values. However, this requires expanding the scope of research.

Analysing the effect of the feed per tooth on the chip temperature, there is also a tendency for the course of the analysed graphs to be similar depending on the tool geometry. In the case of the tool angle λ_s_ = 50°, as the feed increases, the chip temperature decreases proportionally, which justifies the change in the previously mentioned chip thermal capacity. However, in the case of the angle λ_s_ = 20°, it is possible to clearly indicate the feed value from which the temperature stabilizes.

The effect of high chip temperature for low feed is not widely confirmed in the literature. In refs. [20,23,24], this effect was not observed at all, while in the case of Ref. [21] a similar trend can be observed for the AZ91HP alloy.

The obtained results confirm the need to avoid low feed speeds for the sake of process safety. Higher feed rates stabilize or lower chip temperature.

In the case of axial depth of cut, a sharp increase in chip temperature was observed only up to a_p_ = 1.5 mm. Further increasing a_p_ only slightly affects the temperature increase. In all reference studies [20,21,23,24], an increasing trend in temperature was observed with increasing cutting speed. In the case of the cutter helix angle λ_s_ = 50° for axial depth of cut a_p_ above 3 mm, the obtained temperatures were higher for both tested magnesium alloys. For larger axial depths of cut, the chip temperature is relatively easy to predict, which translates into machining safety.

In light of the above, it seems reasonable that future studies should investigate not only maximum chip temperatures but also temperatures that were most frequent in a given test. For safety reasons, the authors focused on the trends in temperature changes depending on changes in cutting parameters because the heat generated during cutting may cause ignition of magnesium chips. Nevertheless, drawing conclusions from a small number of tests may be insufficient to provide an insight into the nature of chip temperature variations. The results led the authors to conclude that it would be reasonable to analyse data manually, as was done in previous studies, and thereby compare the two approaches. Such comparative analysis could be used to develop a more sophisticated procedure for thermographic data analysis. Another direction the authors intend to pursue is the acquisition of thermal data with a thermal imaging system and a thermocouple at the same time in order to compare temperatures measured with the infrared camera to those taken with the thermocouple attached to the workpiece. Data obtained in this manner could be converted into temperature values considering the real emissivity coefficient.

This study is preliminary to further research on chip temperature in the cutting zone in milling processes for magnesium alloys conducted using tools with different cut-ting edge geometries. Future studies on this problem will investigate finishing and precision machining and could concern measurements with a natural thermocouple for preliminary calibration of a thermal imaging camera. An equally interesting aspect of future studies could be the use of FEM to predict chip temperature in the milling process for in-depth analysis of decohesion and accompanying phenomena occurring in the cutting zone as well as for determination of the effect of cutter micro- and macrogeometries on chip temperature.

## 5. Conclusions

The results of this study lead to the following general conclusions:

- The aim of the work, which was to analyse the influence of helix angle and technological parameters on chip temperature in the cutting area, was achieved.
- A significant influence of the cutter helix angle on the relationship between chip temperature and cutting parameters was confirmed.
- This study proposed a new approach to estimating chip temperature in the cutting zone and ensuring safety in magnesium alloy machining.
- The proposed methodology of conducting infrared measurements and processing obtained results was found to be effective in rough milling of magnesium alloys with the use of carbide tools, particularly for variable cutting speed v_c_. Further research must be conducted to investigate the effect of the variables f_z_ and a_p_ because of the following two situations: (a) an increased section of the cutting layer resulted in a chip temperature drop in the cutting zone (for f_z_) and (b) the analysis was performed using different temperature estimation procedures and the processed results were obtained by different algorithms (for a_p_).
- Dry rough milling of magnesium alloys can be performed at higher cutting speed without chip ignition risk (no characteristic type of chip ignition was detected in the experiments).
- For the variable cutting speed v_c_, the maximum chip temperature ranged approx. 297.48 °C–362.95 °C, and no chip ignition was observed during the milling process.
- For the variable feed per tooth f_z_, the maximum chip temperature ranged from approx. 291.18 °C–417.74 °C, and—like in the case of variable v_c_—no chip ignition was observed during the milling process.
- For the variable axial depth of cut a_p_, the maximum chip temperature ranged from approx. 83.17 °C–358.13 °C, and—like in the case of variable v_c_ and f_z_—no chip ignition was observed during the milling process.
- It was difficult to unanimously establish whether the chip temperature in the cutting zone decreased or increased with the variable helix angle and the above milling parameters.
- The highest chip temperature in the cutting zone was obtained for feed per tooth f_z_ = 0.05 mm/tooth and AZ31B and a helix angle of λ_s_ = 50°, and its value was approx. 418 °C.
- The highest observed chip temperature value does not exceed 500 °C (as the minimum temperature for the so-called contractual ignition point). Therefore, the processing can be considered safe.
- Although it was stated that there was no risk of chip ignition within the tested range of milling parameters, it should be noted that appropriate safety procedures should always be followed when machining magnesium alloys.
- The research presented in this paper should be considered preliminary; the proposed research methodology can be employed to conduct the full range research for all machining parameters.

## Figures and Tables

**Table 1:** Compilation of selected research results on the machinability of magnesium alloys.

Grade of Magnesium Alloy	Machining Conditions	Research Object	Reference
AZ91HP	Milling (carbide end mill): a_p_ = 1 mm, f_z_ = 0.055 mm/tooth, v_c_ = 35–105 m/min, a_e_ = 5 mm	Workpiece temperature	[2]
AZ91	Milling (micro-grain tungsten carbide ball-nose end-mills): v_c_ = 408–1088 m/min v_f_ = 50–7000 mm/min a_p_ = 0.05–3.0 mm Cooling and lubricating Dry	Mean flank temperature, undeformed chip thickness, SEM chips images	[9]
Mg-Ca0.8	High-speed dry milling (milling cutter with PCD tipped inserts): v_c_ = 1200–2800 m/min f = 0.05–0.4 mm/rev a_p_ = 0.1–0.5 mm	Temperature (tool/chip interfaces (across chip) and in the subsurface), roughness Ra parameter, principal residual stresses, subsurface microstructures	[10]
Mg-Ca0.8	High-speed dry milling (milling cutter with PCD tipped inserts): v_c_ = 1200–2800 m/min f = 0.05–0.4 mm/rev a_p_ = 0.1–0.5 mm	SEM chip morphology, flank face adhesion, surface roughness Ra values, cutting and thrust force, temperature distribution	[11]
AZ91C	High-speed milling (HSS end mill): v_c_ = 37.6–680 m/min v_f_ = 120 mm/min a_p_ = 0.5–1.0 mm	Temperature (on the machining zone and work-piece temperature)	[12]
AM50A	Face milling (milling carbide cutter with K110M inserts): v_c_ = 151–3014 m/min f_z_ = 0.02 mm/tooth a_p_ = 15 µm a_e_ = 60 mm Cooling and lubricating dry	Mean flank temperature, SEM micrographs of tool tip and chip surface morphology, cutting force F_x_	[13]
AM50A and AZ91D	Face milling (milling carbide cutter with K110M inserts): v_c_ = 754 m/min v_f_ = 400 mm/min a_p_ = 5–45 µm a_e_ = 60 mm Cooling and lubricating dry	Images of ignition conditions at different depth of cut, morphology of chips	[14]
AM50A	Face milling (milling carbide cutter with K110M inserts): v_c_ = 251–1507 m/min v_f_ = 100–1000 mm/min a_p_ = 1–1000 µm a_e_ = 55 mm Cooling and lubricating dry	Chip ignition conditions, morphologies and SEM images	[15]
AZ91HP, AZ31	High-speed dry milling (carbide end mill with TiAlN coating, carbide Kordell geometry end mill, end mill with PCD blade): a_p_ = 0.5–6 mm, f_z_ = 0.05–0.3 mm/tooth, v_c_ = 400–1200 m/min, a_e_ = 14 mm	Temperature in the cutting area (embedded thermocouples, optical pyrometry, infrared measurements)	[20]
AZ91HP, AZ31	High-speed dry milling (carbide end mill with TiAlN coating): a_p_ = 0.5–6 mm, f_z_ = 0.05–0.3 mm/tooth, v_c_ = 400–1200 m/min, a_e_ = 14 mm	Chip temperature in the cutting area	[21]
AZ91HP, AZ31	High-speed dry milling (carbide end mill with TiAlN coating): a_p_ = 0.5–6 mm, f_z_ = 0.05–0.3 mm/tooth, v_c_ = 400–1200 m/min, a_e_ = 14 mm	Time to ignition, ignition temperature, metallographic chips photographs	[22]
AZ91HP, AZ31	High-speed dry milling (end mill with PCD blade): a_p_ = 0.5–6 mm, f_z_ = 0.05–0.3 mm/tooth, v_c_ = 400–1200 m/min, a_e_ = 14 mm	Chip mass, chip temperature in the cutting area, metallographic photographs of chips, successive stages preceding chip ignition	[23]
AZ91HP, AZ31	High-speed dry milling (carbide Kordell geometry end mill): a_p_ = 0.5–6 mm, f_z_ = 0.05–0.3 mm/tooth, v_c_ = 400–1200 m/min, a_e_ = 14 mm	Chip temperature in the cutting area	[24]

**Table 2:** Temperatures of AZ31B obtained in a cutting process conducted with v_c_ = 400 m/min, λ_s_ = 20°, ε = 0.31.

Median °C	Average °C	Max Temp. °C	Min Temp. °C	Range	Std. Deviation
286.43	285.90	307.88	257.42	50.47	9.001

**Table 3:** Temperatures of AZ31B obtained in a cutting process conducted with v_c_ = 1200 m/min, λ_s_ = 20°, ε = 0.31.

Median °C	Average °C	Max Temp. °C	Min Temp. °C	Range	Std. Deviation
316.63	314.65	336.97	276.41	60.56	12.21

**Table 4:** Temperatures obtained from Tests 1 through 9 for different materials and helix angles λ_s_, with emissivity coefficients of ε = 0.31 for AZ31B and ε = 0.24 for AZ91D.

v_c_ (m/min)	T_max_ (°C)	Workpiece	λ_s_	v_c_ (m/min)	T_max_ (°C)	Workpiece	λ_s_
400 500 600 700 800 900 1000 1100 1200	307.88 297.48 304.66 312.53 315.26 314.85 318.66 328.29 336.97	AZ31B	λ_s_ = 20°	400 500 600 700 800 900 1000 1100 1200	340.30 340.22 344.37 354.27 351.32 340.16 341.25 341.34 336.50	AZ31B	λ_s_ = 50°
400 500 600 700 800 900 1000 1100 1200	325.62 321.32 331.31 345.16 362.95 355.26 358.40 360.85 314.72	AZ91D	λ_s_ = 20°	400 500 600 700 800 900 1000 1100 1200	348.11 351.00 347.23 339.90 343.96 337.15 330.62 325.88 322.60	AZ91D	λ_s_ = 50°

**Table 5:** Temperatures of AZ31B alloy obtained in a cutting process conducted with f_z_ = 0.05 mm/tooth, λ_s_ = 20°, ε = 0.31.

Median °C	Average °C	Max Temp. °C	Min Temp. °C	Range	Std. Deviation
350.02	351.02	395.86	279.53	116.32	20.35

**Table 6:** Temperatures obtained from Tests 10 through 15 for different materials and helix angles λ_s_, with an emissivity coefficient ε = 0.31 for AZ31B and with an emissivity coefficient of ε = 0.24 for AZ91D.

**f_z_ (mm/tooth)**	**T_max_ (°C)**	**Workpiece**	**λ_s_**	**f_z_ (mm/tooth)**	**T_max_ (°C)**	**Workpiece**	**λ_s_**
0.05 0.1 0.15 0.2 0.25 0.3	395.86 335.59 311.57 319.00 318.66 312.45	AZ31B	λ_s_ = 20°	0.05 0.1 0.15 0.2 0.25 0.3	413.35 368.37 341.83 315.06 296.07 268.55	AZ31B	λ_s_ = 50°
0.05 0.1 0.15 0.2 0.25 0.3	410.77 359.48 358.05 363.43 361.63 361.63	AZ91D	λ_s_ = 20°	0.05 0.1 0.15 0.2 0.25 0.3	417.74 360.48 339.57 305.26 291.19 291.18	AZ91D	λ_s_ = 50°

**Table 7:** Temperatures of AZ31B alloy obtained in a cutting process conducted with a_p_ = 0.5 mm, λ_s_ = 20°, ε = 0.31.

Median °C	Average °C	Max Temp. °C	Min Temp. °C	Range	Std. Deviation
66.04	70.19	158.30	37.32	120.97	19.99

**Table 8:** Temperatures obtained from Tests 16 through 21 for different materials and helix angles λ_s_ with an emissivity coefficient of ε = 0.31 for AZ31B and an emissivity coefficient of ε = 0.24 for AZ91D.

**a_p_ [mm]**	**T_max_ [°C]**	**Workpiece**	**λ_s_**	**a_p_ [mm]**	**T_max_ [°C]**	**Workpiece**	**λ_s_**
0.5 1 1.5 3 4.5 6	158.30 199.16 281.27 298.42 301.34 313.10	AZ31B	λ_s_ = 20°	0.5 1 1.5 3 4.5 6	217.63 232.13 264.63 313.96 358.13 341.70	AZ31B	λ_s_ = 50°
0.5 1 1.5 3 4.5 6	227.18 241.85 267.93 296.52 308.87 318.93	AZ91D	λ_s_ = 20°	0.5 1 1.5 3 4.5 6	83.17 228.82 273.41 331.74 339.70 343.49	AZ91D	λ_s_ = 50°

## Data Availability

The data presented in this study will be available on request from the corresponding author. The data are not publicly available due to restrictions of the study being on-going.
